# Neuroinflammation and Neuroimmune Dysregulation after Acute Hypoxic-Ischemic Injury of Developing Brain

**DOI:** 10.3389/fped.2014.00144

**Published:** 2015-01-14

**Authors:** Utpal S. Bhalala, Raymond C. Koehler, Sujatha Kannan

**Affiliations:** ^1^Department of Anesthesiology, Johns Hopkins University School of Medicine, Baltimore, MD, USA; ^2^Department of Critical Care Medicine, Johns Hopkins University School of Medicine, Baltimore, MD, USA

**Keywords:** hypoxia-ischemic encephalopathy, inflammation, developing brain, microglia, immune dysregulation

## Abstract

Hypoxic-ischemic (HI) injury to developing brain results from birth asphyxia in neonates and from cardiac arrest in infants and children. It is associated with varying degrees of neurologic sequelae, depending upon the severity and length of HI. Global HI triggers a series of cellular and biochemical pathways that lead to neuronal injury. One of the key cellular pathways of neuronal injury is inflammation. The inflammatory cascade comprises activation and migration of microglia – the so-called “brain macrophages,” infiltration of peripheral macrophages into the brain, and release of cytotoxic and proinflammatory cytokines. In this article, we review the inflammatory and immune mechanisms of secondary neuronal injury after global HI injury to developing brain. Specifically, we highlight the current literature on microglial activation in relation to neuronal injury, proinflammatory and anti-inflammatory/restorative pathways, the role of peripheral immune cells, and the potential use of immunomodulators as neuroprotective compounds.

## Introduction

Regions of the developing brain become vulnerable to hypoxic-ischemic (HI) as the neurons develop operating voltage-dependent ion channels, neurotransmitter receptors, synaptic connections, and increased mitochondria to supply the ATP for the consequent increase in energy demand (1). Global HI injury in developing brain can result from a variety of clinical conditions, including perinatal asphyxia and cardiac arrest in infants and older children. Although rates of survival to hospital discharge from in-hospital cardiac arrest in children has improved significantly over the last decade, neurologic outcomes remain poor (2).

A cascade of cellular and biochemical responses to the initial HI insult can lead to secondary neuronal injury after reoxygenation. One of the crucial but understudied mechanisms of secondary neuronal injury after global HI is inflammation (3). It is characterized by activation of microglia, the innate immune cells of brain, migration of peripheral macrophages; release of proinflammatory cytokines and chemokines, and phagocytosis of injured and uninjured neurons. Some evidence suggests that blocking the inflammatory reaction promotes neuroprotection and has potential for use in the clinical treatment of ischemic brain injury (4–8).

Therapeutic hypothermia has been shown to protect the brain after cardiac arrest in adults and after HI in term newborns (9–11); it is currently undergoing a multicenter trial for children after cardiac arrest (12). Although therapeutic hypothermia reduces mortality and improves early neurologic outcome after HI injury in neonates, significant neurologic deficits and learning disability persist into childhood (13, 14). Cell death initiated before the onset of hypothermia, or possibly after rewarming, will likely recruit inflammatory processes that can contribute to the low efficacy of hypothermia in newborns who experience the most severe HI insult. In a multicenter, randomized trial of induced hypothermia for neonatal hypoxic-ischemic encephalopathy (HIE), a rapid and dramatic rise in levels of proinflammatory cytokines immediately after HI injury was not amenable to induced hypothermia (15). Adjunct treatment targeted to inflammation and immune dysregulation may help improve the overall efficacy of therapeutic hypothermia (16).

In this article, we review current understanding about the inflammatory and immune mechanisms involved in acute HI injury of developing brain and the neuroprotective agents that can curtail inflammation and immune dysregulation. New anti-inflammatory targets continue to be identified and constitute an important area for translational medicine (17–22). Overall, the prospects for safe neuroprotective therapies to improve outcome after acute HI brain injury remain promising.

## HI Neuronal Injury in Developing Brain

A wealth of information on HI brain injury and repair is available through translational adult stroke research. The immature brain differs from the adult in its capability to use metabolic fuels, vulnerability to glutamate excitotoxicity, and oxidative stress (1). There is an evidence to suggest that age could have a significant effect on response to cytokines and hence neuroinflammation after exposure to lipopolysaccharide (LPS) and/or HI (23). Since the mechanisms of injury and strategies for repair often are very different in the immature as compared with the adult brain, our pediatric community and researchers need to focus on HI brain injury and repair in the developing brain (24, 25).

Global HI of brain initiates a cascade of excitotoxicity and oxidative damage that in turn causes microvascular injury, blood–brain barrier (BBB) dysfunction, and postischemic inflammation. These events all exacerbate the initial injury and can lead to permanent cerebral damage. The modes of secondary neuronal loss after HI are apoptosis, autophagy, programed necrosis, and unregulated necrosis, which arise from cell swelling and bursting of nuclear and cell membranes (26). Apoptosis is more prominent in the neonatal brain than in the adult brain after HI insult (27–29). Also, selective neuronal vulnerability has been observed in developing brain whereby neuronal injury secondary to HI is present predominantly in regions that function in sensorimotor integration and movement control. The regions in the developing brain, which are vulnerable to HI are sensory-motor cortex, basal ganglia, thalamus, and hippocampus (30). Considerable research has been targeted toward different pathways of secondary neuronal injury and therapies to counter one or more such pathways.

## Inflammation and Immune Dysregulation after Acute Insult to the Brain

For many years, the brain was considered an immune-privileged organ, but advances in neuroimmunology have challenged this dogma and helped to expand our understanding of the immune processes that occur in healthy and diseased brain (31). It is well accepted that the brain and immune system are engaged in bidirectional crosstalk. Microglia, the resident innate immune cells of the brain, elicit inflammatory responses under neuropathologic conditions such as perinatal HI encephalopathy, infection, and traumatic brain injury, as well as in autoimmune and neurodegenerative disorders (32). Also, growing evidence indicates that, like in peripheral organs, inflammatory cells play a crucial role in remodeling and repair after an acute insult to the brain. The neuroinflammatory response after acute HI brain injury is characterized by activation of microglia; migration of peripheral macrophages, monocytes, and neutrophils; and release of cytokines and chemokines by the inflammatory cells (33).

## Microglia – Innate Immune Cells of Brain

Microglia are resident macrophages of brain and are known to actively remove cellular debris during normal development and under pathologic conditions. They were first identified as innate cells of the central nervous system (CNS), distinct from neurons and other glia, by Nissl in 1899. Many years later, in the early part of the 20th century, del Rio Hortega confirmed this distinction using silver carbonate staining methods (34) and proposed that these cells were primarily of hematopoietic origin. Microglia constitute 10–15% of the total glia within the brain (35) and are present throughout the brain. Some areas are more heavily populated than others, and white matter generally contains fewer microglial cells than does gray matter (36). Microglia are highly ramified cells, and under non-pathologic conditions, they have a small cell body with long and densely branched processes.

## Role of Microglia in Developing Brain

Although microglia are highly active during pathologic conditions, they are also active under physiologic conditions as they survey the microenvironment and the status of neurons and participate in housekeeping and remodeling within the CNS (37). In developing brain, highly active neurogenesis forms large numbers of neurons every minute, vastly exceeding the ultimate requirements of an adult. Consequently, many neurons undergo apoptosis during early infancy and childhood (38). Microglia have a functional role in the phagocytosis of cell debris and in the release of trophic factors in developing brain. They also are involved with synaptic pruning in the developing brain, thereby influencing its maturation (37, 39–42).

## Role of Microglia in HI Injury of Developing Brain

Microglia fulfill a variety of tasks after HI of the brain. They engulf cellular debris, lipids, and apoptotic cells. They also promote cytotoxicity through release of proteases and proinflammatory cytokines, activation of respiratory burst, and *N*-methyl-d-aspartate (NMDA)-mediated excitotoxicity. These mechanisms not only scavenge the HI damaged neurons but they can also affect viable neurons. Much of our current knowledge of inflammation, particularly microglial activation, after HI injury of developing brain comes from the Vannucci model, in which HI is induced in postnatal rats and mice by exposure to low oxygen and carotid ligation (43, 44).

### Time course of microglial activation after HI

Hypoxia–ischemia and intra-cerebral administration of excitotoxins such as NMDA result in a fast and robust microglial reaction in the developing brain (45). One of the earliest studies of microglial activation after HI brain injury in a rat model of developing brain described microglial activation within 2 h after HI injury (6). In another study, microglia exhibited a time-dependent, differential upregulation of MHC and CD4/CD8 immunomolecules from day 1 to day 28 post-injury (46).

Whereas early neuronal cell death undoubtedly activates microglia, a key question is whether activated microglia contribute to delayed cell death of other neurons. Co-culture studies of microglia and oxygen-glucose-deprived neurons have shown that stressed neurons activate microglia, which release proinflammatory cytokines. These cytokines then induce neuronal damage (47). However, uncertainty persists about the extent to which neuronal and non-neuronal cells communicate *in vivo* as they do *in vitro*, especially under pathologic conditions involving multiple cell types. Nevertheless, the concept that the proinflammatory cytokines released by active microglia contribute to ongoing secondary neuronal injury has gained support from the neuroprotective effects seen with therapies targeting microglial activation (4–8, 17–22).

Little is known about the morphologic and functional changes that microglia undergo over time after acute, global, HI developmental brain injury, or about the regional distribution of active microglia in relation to selective neuronal vulnerability. Also, the duration of the proinflammatory response beyond the period of acute injury is not clearly defined. Acute inflammation can also be shifted to a chronic inflammatory state and/or adversely affect brain development (48).

Activated microglia play an equally important role in the restorative and reparative processes after neuronal injury (49). Neuroprotective therapies targeted toward microglia could potentially be a double-edged sword if used without appropriate information on time course of the proinflammatory and restorative responses after acute, global HI.

### Microglial activation and white-matter injury after HI

Apart from secondary neuronal injury after HI, microglia have been found to play a crucial role in oligodendrocyte injury and disturbance in myelination through a cytokine-mediated mechanism in neonatal hypoxic rats (50). Likewise, overproduction of local cytokines by activated microglia has been reported to induce axonal injury after hypoxia in developing rats (51). Microglia contribute to white mater injury in immature, developing brain (52). In studies of perinatal brain injury, intrauterine inflammation has shown to cause white-matter injury through microglial activation (53). Perinatal HI injury in preterm brain is associated with a T-helper-type immune response (54). Perinatal inflammation, which triggers neuroinflammation is also believed to predispose the immature brain to HI injury (55, 56).

### Microglial activation and BBB integrity after HI

Under normal conditions, the BBB is important for maintaining micro-environmental homeostasis in the brain and its so-called immune-privileged status by preventing the entry of T lymphocytes (57, 58). In a combined *in vivo* and *in vitro* study of the relationship between microglial activation and BBB under HI conditions, increased BBB disruption was associated with activated microglia. However, this association was inhibited by the anti-inflammatory action of minocycline, which is known to inhibit the release of matrix metalloproteinase (MMP)-9 and breakdown of collagen and laminin in the vascular basement membrane (59).

### Microglial proliferation and migration after injury

Microglia have a remarkable ability to multiply and migrate in response to neurologic injury (60, 61). Microglial proliferation has been implicated in the onset and/or progression of ischemic brain injury (62). Active microglia express receptors for a variety of molecules, such as interleukin (IL)-3, IL-6, and granulocyte-macrophage colony-stimulating factor, which play an important role in microglial proliferation (63). *In vivo* studies of the cell proliferation markers Ki67 and bromodeoxyuridine have confirmed that microglia can proliferate in a developing brain environment (64). A rapid increase in number of microglia at the site of injury is related to influx of peripheral monocytes and movement of innate microglia from other parts of the brain (65).

## Role of Astrocytes

Astrocytes express a wide variety of receptors of innate immunity (66). In response to HI, there is reactive astrogliosis with release of MMPs, which degrade BBB and facilitate entry of peripherally derived immune cells (67, 68). Through toll-like receptors, astrocytes, on one hand promote inflammation and on the other end, facilitate tissue repair (66, 69). In response to ischemia, astrocytes not only potentiate excitotoxicity through inducible nitric oxide synthase (iNOS) but also release a myriad of cytokines, many of which have dual proinflammatory and anti-inflammatory effects (70–74).

## Role of Proinflammatory and Anti-Inflammatory Cytokines and Chemokines after HI Injury of Developing Brain

**Figure 1:**
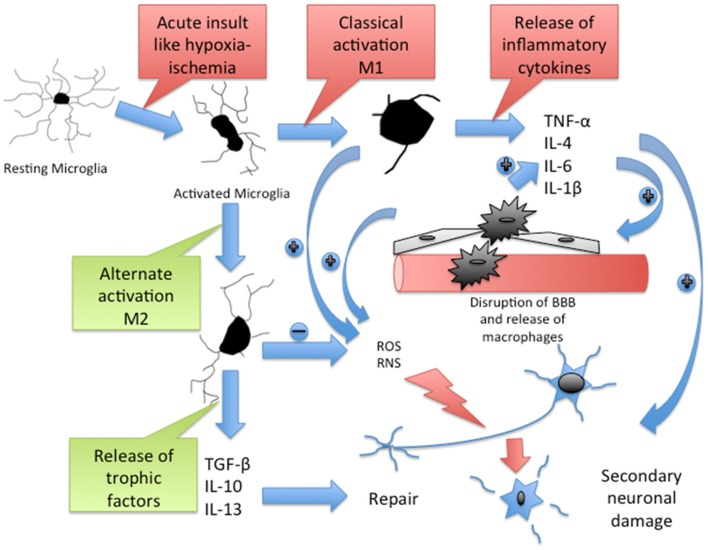
**Cascade of inflammatory pathway in brain after acute HI**. Resting microglia get activated to M1 type through classical pathway and M2 type through alternate pathway. M1 microglia release inflammatory cytokines, which cause disruption of blood–brain barrier (BBB). The BBB disruption promotes infiltration of macrophages, which further release inflammatory cytokines. Active microglia and macrophages release reactive oxygen species (ROS) and reactive nitrogen species (RNS), which contribute to the secondary neuronal injury. M2 microglia release trophic factors, which contribute to the neuronal repair. TNF-α, a potent proinflammatory cytokine contributes to peripheral immune cell recruitment and proliferation in the brain, neuronal apoptosis, oligodendrocytes, and axonal injury after HI (94). IL-1β blocks oligodendrocyte proliferation (95) and elevates levels of circulating IL-6, another potent cytokine that contributes to early neurologic deterioration after brain ischemia (95, 96). Matrix metalloproteinases (MMPs) disrupt the BBB and allow peripheral leukocyte infiltration (97). Macrophage colony stimulating factor (MCSF), released mainly by macrophages, T cells, B cells, and microglia, induces proliferation, migration, and activation of microglia and regulates the release of proinflammatory cytokines from macrophages (98, 99). MCP-1, a chemokine secreted by active microglia and astrocytes in response to injury (100), mediates the migration of microglia, monocytes, and lymphocytes to the site of injury in the CNS (101, 102).

Cytokines and chemokines released by active microglia in response to an acute neurologic insult take part in innate immune response; modulate influx of peripheral immune and inflammatory cells into the brain; contribute to secondary neuronal, oligodendrocyte, and axonal injury; and ultimately promote tissue repair and recovery (75) (Figure 1). In the brain, cytokines and chemokines are expressed on the neurons and glia (76). There is increased chemokine gene expression and release in the developing brain after HI (77). Cytokines and chemokines released by peripheral immune cells contribute to neuroinflammation (78, 79) and their inhibition or deficiency is associated with reduced injury (80–82). TRAIL (tumor necrosis factor-related apoptosis inducing ligand) is expressed primarily on microglia and astrocytes and it has been shown to participate in neonatal brain injury after inflammation and HI (83). Elevated levels of IL-6 and IL-8 in the cerebrospinal fluid of term newborns have been correlated with an increased degree of encephalopathy and poor neurodevelopmental outcome (84). Reactive oxygen species (ROS) and nitrogen metabolites generated within the active microglia induce the release of proinflammatory cytokines. In a study of mixed astroglial/microglial cultures, stimulated microglia produced NO in a time-dependent manner (85). Following HI injury, hydrogen peroxide (H_2_O_2_) levels rise significantly (86) and cause extensive damage to iron-rich developing brain (87). In fact, microglial exposure to continuous H_2_O_2_ leads to pleiotropic and biphasic effects (88). Since the effects of cytokines are influenced by one another and majority of cytokines have pleiotropic and cell-specific effects, the final effect of individual cytokines is difficult to establish (89–93).

## Microglial Activation after Ischemia – Harmful or Helpful?

Microglia function as CNS macrophages and help clear debris and invading pathogens. When activated in response to a variety of stimuli and triggering events such as HI, proinflammatory and cytotoxic pathways are initiated that can contribute to secondary neuronal injury. Conversely, stimulation of proliferating microglia after cerebral ischemia by MCSF leads to release of insulin-like growth factor (IGF), a neurotrophic factor with neuroprotective properties (103). In a rodent model of focal cerebral ischemia, time-lapse imaging showed that microglia exert neuroprotection by rapidly engulfing apoptotic neurons and motile polymorphonuclear cells (104). In a study of rodent cerebral ischemia, intra-arterial injection of microglia prevented the ischemia-induced decline of brain-derived neurotrophic factor (BDNF) in hippocampus and offered neuroprotection (105). Similarly, in a rodent model of acute neonatal stroke, inhibition of microglia with liposomal clodronate led to elevation of levels of cytokines and chemokines and exacerbation of injury (103). There is growing evidence that microglia activated by injured or dying neurons mediate a decrease in neuronal damage and promote tissue regeneration and repair (106). In response to injury, activated microglia ensheath damaged neurons and remove excitatory input through the displacement of afferent synapses (107). Much of our current understanding of the difference between helpful and harmful microglial phenotypes is derived from literature on acute neuroinflammation after stroke and chronic neuroinflammatory conditions like Alzheimer’s disease (108, 109). The innate immune response is characterized by activation of microglia to an M1 phenotype and the subsequent proinflammatory response followed by resolution, and alternative activation to an M2 phenotype that leads to anti-inflammatory signaling (M2a), the clearance of ROS and reactive nitrogen species (RNS) (M2b), and wound healing (M2c) (110). Depending on the type of insult, the phenotype of microglial activation switches over time from M1 to M2 or vice versa (111). The differences between M1 and M2 microglial phenotypes are shown in Table 1. The M1 phenotype is associated with greater neuronal death than is the alternatively activated M2 phenotype (112); therefore, there is a growing interest in inhibiting the M1 phenotype.

**Table 1:** **The M1 (classical) and M2 (alternate) phenotypes of microglia**.

Classical activation (M1)		Alternative activation (M2)	
Identification markers	Proinflammatory cytokines	Identification markers	Anti-inflammatory cytokines
MHCII	IFNγ	Arg-1	IL-10
CD16 (FcγR III)	I1-1β	CD68 (ED1)	TGF-β
CD32 (FcγR II)	TNFα	Fizz1 (Relmα)	IL-4
CD80 (B7-1)	I1-6	Ym-1	IL-13
CD86 (B7-2)	CXCL10	CD206 (MR)	IGF-1
CD40 (TNFR)	ROS	Dectin-1	
	RNS		
	MMP9		
	MMP3		

*Arg, arginase; Fizz1, resistin-like molecule alpha; IFN, interferon; IL, interleukin; MHC, major histocompatibility complex; MMP, matrix metalloproteinase; MR, mannose receptor; RNS, reactive nitrogen species; ROS, reactive oxygen species; TGF, transforming growth factor; TNFR, tumor necrosis factor receptor*.

## Regulation of Microglia

Microglia are kept under check through neuronal–glial cross talk. Chemokine receptors and corresponding ligands allow interactions between neurons and microglia and control proinflammatory responses of microglia under physiologic conditions (113). CD200/CD200R1, fractalkine (CX3CL1)/CX3CR1, SIRPα/CD47, and heat shock protein 60 (HSP60)/TREM2 are cell–cell interaction molecules that regulate microglia (114–117). After injury, disruption of these interactions from neuronal damage may cause activation of microglia to the proinflammatory M1 phenotype. Modifications of these neuronal–glial regulatory interactions have been shown to attenuate neuronal damage in models of focal cerebral ischemia and chronic neuroinflammation (118–122). Neuronal–glial interactions and their role in secondary neuronal injury after global HI injury in developing brain are understudied and warrant evaluation. Modifications of these regulatory interactions in developing brain after global, HI injury could potentially open new avenues of neuroprotection.

## Role of Peripheral Inflammatory and Immune Cells in the Developing Brain after HI

**Figure 2:**
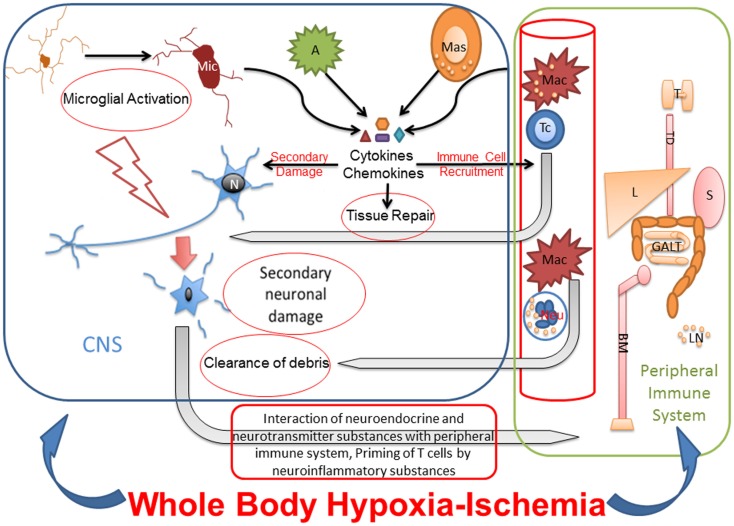
**Whole-body hypoxia-ischemia activates central and peripheral immune components**. The whole-body HI not only induces neuroinflammation and necrosis and apoptosis in brain but also potentially exerts different effects in peripheral immune organs like spleen, which is influenced by the autonomic nervous system. Microglial activation after global HI leads to secondary neuronal injury. The cytokines and chemokines released by activated microglia, astrocytes, mast cells, and peripheral immune cells cause secondary neuronal damage, degrade BBB for leukocyte recruitment from blood stream, and eventually also contribute to tissue repair. Primed and polarized T cells enter CNS in response to HI, recognize neuronal surface receptors like major histocompatibility complex, and interact with damaged neurons for repair. Macrophages and neutrophils also enter the CNS in response to HI for clearance of debris. Under pathologic conditions, neurotransmitter and neuroinflammatory substances interact with peripheral immune system for priming and activating immunologic pathways for clearance of pathogen and/or debris. Due to cross talk between peripheral and central immune systems, the effects of peripheral immune organs like thymus, liver, spleen, gut associated lymphoid tissues and bone marrow on neuroinflammation, and secondary neuronal injury after whole-body HI need to be studied. Mic, microglia; N, neuron; A, astrocyte; Mas, mast cell; Mac, macrophage; Tc, T cell; Neu, neutrophil; T, thymus; TD, thoracic duct; L, liver; S, spleen; GALT, gut-associated lymphoid tissue; BM, bone marrow.

Studies from adult rodent models of focal HI suggest that, in addition to innate inflammation, the peripheral immune system may have a role in the etiology of neuronal damage. More precisely, several studies have shown that acute brain injury from focal ischemia is associated with a massive activation of the peripheral immune system, with rapid mobilization of immune effector cells from the spleen (123, 124). These mobilized effector cells can invade the brain and aggravate the existing injury (123). In a study of preterm sheep in which global HI was induced with the umbilical cord occlusion method, the authors showed mobilization of peripheral immune cells from spleen and reduction of splenic size. These changes were unrelated to splenic HI injury and occurred in parallel with marked injury and functional loss of the preterm brain (125). Yilmaz and co-authors showed that T lymphocytes, but not B lymphocytes, contribute to inflammatory and thrombogenic responses, brain injury, and neurologic deficit associated with experimental stroke in rodents (126). Though studies have shown that activated microglia outnumber the peripherally derived macrophages at the site of infarct (127, 128), inhibition of macrophages has shown to reduce infarct volume (129–131). These reports indicate that infiltrating mononuclear inflammatory cells play a significant role in neuroinflammation and may be necessary for the activation of microglia. Suppression of peripheral immune and inflammatory cells through splenectomy reduces cellular infiltration into the brain and interaction with the activated microglia at the site of ischemic injury, resulting in decreased damage (123). A severe, global HI injury following cardiac arrest would not only induce necrosis and apoptosis in brain but also might exert different effects in peripheral immune organs like spleen, which is influenced by the autonomic nervous system. Therefore, additional studies are warranted to determine the role of the peripheral immune system in whole-body HI (Figure 2).

## Anti-Inflammatory and Immunomodulatory Therapies for Neuroprotection after HI Brain Injury

Preconditioning, salvaging, and repair are three main modes of achieving neuroprotection (132).

Hypothermia is now standard of care for term HI encephalopathy, so studies focused on adjunct therapies will be added to that treatment (133). As our understanding has grown regarding the role that inflammatory and immune cells play in the pathophysiology of secondary neuronal injury after HI, so has interest in using anti-inflammatory and immunomodulatory strategies as neuroprotective therapies after brain injury. Such therapies range widely from steroidal and non-steroidal molecules to cannabinoids (CB) and statins.

### Cannabinoids

Cannabinoids are diverse chemical compounds, which are either endogenously produced in body (endocannabinoids) or derived from cannabis and related plants (phytocannabinoids) or prepared chemically (synthetic cannabinoids). CB compounds act on cell surface CB receptors to exert a variety of effects, including potent anti-inflammatory and immunomodulatory effects. There are two major subtypes of CB receptors – CB1 and CB2. The CB1 receptors present on neurons produce the psychoactive effects of non-selective CBs like tetrahydrocannabinol (134). CB2 receptors are present on immune cells (135) and are expressed on the surface of activated microglial cells under pathologic conditions (136–138). Under physiologic state, CB2 receptors remain dormant, and they are expressed in the active form on the surface of the immune cells after an acute insult. CB2 receptor agonists such as cannabidiol (CBD) exert potent anti-inflammatory and immunomodulatory actions through a CB2 receptor-G-protein-coupled mechanism (139, 140). Two orphan G-protein-coupled receptors, possibly activated by multiple different cannabinoid ligands, have been recently proposed as novel cannabinoid receptors (141).

Cannabidiol is a major constituent of the cannabis plant, representing up to 40% in plant extracts. CBD has shown neuroprotective effects in adult rodent models of stroke (142–144). CBD studies have also shown significant neuroprotective effects in a piglet model of HI (17–21). CB2 agonists reduce microglial activation, proliferation and migration to the site of injury, and also reduce release of proinflammatory cytokines and chemokines like IL-1β, TNFα, MCP-1, and MIP-1α (20). The neuroprotective effects of CBD are related not only to the anti-inflammatory and immunomodulator effects but also to serotonergic, antiexcitotoxic, antioxidant, adenosine receptor agonist, and antiepileptic effects (18–21). Due to multi-pronged neuroprotective effects, including but not limited to anti-inflammatory and immunomodulatory effects, CB have gained recent interest in research on developmental brain injury. Because CBD is a selective CB2 receptor agonist and lacks CB1-induced psychoactive effects, the potential for its clinical use seems favorable (145). However, certain crucial questions that need to be addressed in preclinical setting before considering CBD for clinical trials are dose–effect relationship, the role of CB2 agonists as adjunct neuroprotective agents with therapeutic hypothermia, and the role of CB2 agonists as neuroprotective agents through peripheral versus central immunomodulation.

### Anti-TNF-α

TNF-α is a potent proinflammatory cytokine that plays a key role in neurotoxicity after ischemia. Anti-TNF-α has been shown to be neuroprotective in rodent models of focal cerebral ischemia (146, 147). The challenges with the use of anti-TNF-α have been solubility and brain penetration. Additionally, TNF inhibitors can have harmful side effects, including lymphoma, infections (especially tuberculosis reactivation), congestive heart failure, demyelinating disease, and lupus-like syndrome (148).

### IL-1 antagonists

Recombinant human IL-1 receptor antagonist (rhIL-1ra) has been shown to protect against focal cerebral ischemia in the rat through its actions on microglia (149). A randomized phase II study of rhIL-1ra in acute stroke patients showed that it is safe and well tolerated in acute stroke. In addition, rhIL-1ra exhibited biologic activity that was relevant to the pathophysiology and clinical outcome of ischemic stroke (150).

### Minocycline

Minocycline is a tetracycline derivative that has been shown to be safe and effective as an antibiotic and anti-inflammatory drug for treating systemic inflammatory conditions. Minocycline crosses the BBB and has demonstrated neuroprotective qualities in experimental models of post-arrest global HI (7), traumatic brain injury, stroke, spinal cord injury, and neurodegenerative diseases (151). Minocycline is believed to have anti-inflammatory, antiapoptotic, and antioxidant effects. It inhibits microglial activation, T-cell migration, and release of proinflammatory cytokines and chemokines (151). Minocycline administered either immediately before or immediately after a HI insult substantially blocked tissue damage in a rodent model of neonatal HI brain injury (152). In a model of neonatal stroke, minocycline significantly reduced the volume of injury at 24 h but not 7 days after transient MCA occlusion (153). Unfortunately, minocycline has also been shown to have variable and even detrimental effects in different species and models of neurological disorders (151, 154–158). Although its anti-inflammatory actions are likely to contribute to its neuroprotective effects, its contrasting effect in mouse and rat HI models could be related to its reported action on the regulation of prostaglandin pathways (154). Also, long-term minocycline therapy in chronic neuroinflammatory diseases has proved disappointing due to either minimal or no clinical effects, even worse effects, and safety concerns (159–161).

### Ibudilast

Ibudilast is mainly a phosphodiesterase (PDE) inhibitor, but it also affects the function of lymphocytes, endothelial cells, and glial cells. In a neuronal-microglial co-culture study, ibudilast suppressed neuronal necrosis that was induced by LPS and interferon-γ activation of microglia (162). Idibulast acts through phosphodiesterase 4 (PDE4) receptors and inhibits release of TNF- α by inflammatory cells and inhibits tyrosine kinase in neutrophils to mitigate inflammation (163). Currently, there are no clinical trials on neuroproctective effects of ibudilast.

### Vitamin D

Recent evidence supports the involvement of vitamin D3 in immunologic processes that protect the nervous system (164). In the CNS, the active form of vitamin D – calcitriol – acts as an immunosuppressor. It induces the anti-inflammatory cytokine IL-4 and transforming growth factor and decreases expression of proinflammatory cytokines IL-6, TNF, and MCSF (165–169). Calcitriol decreases expression of MHC class II proteins and cofactor CD4, which play important roles in autoimmune processes in the nervous system (170). Role of vitamin D in modulating inflammation and immune dysregulation and ability to offer neuroprotection in a model of global HI have not yet been well studied.

### Steroid and non-steroidal anti-inflammatory drugs

Steroid molecules, including sex steroids, inhibit microglia and prevent release of proinflammatory cytokines. Steroid molecules also offer neuroprotection through release of neurotrophic factors from microglia (171). However, we need more basic information regarding the mechanisms by which steroids contribute to neuroprotection before we can predict the conditions in which hormone treatments may have positive outcomes for brain function in human beings. Such knowledge will enable researchers to design the best possible therapeutic approaches. Non-steroidal anti-inflammatory medications have been shown to curtail neuroinflammation associated with Alzheimer’s disease, Parkinson’s disease, and HI brain injury (170, 172).

### Statins

Statins have been shown to reduce infarct size in experimental animal models of stroke. Statins attenuate the inflammatory cytokine responses that occur after cerebral ischemia, and their antioxidant properties ameliorate ischemic oxidative stress in the brain. Additionally, statins upregulate endothelial nitric oxide synthase and inhibit iNOS, effects that are potentially neuroprotective (173). Currently, NeuSTART2 (neuroprotection with statin therapy for acute recovery trial phase 2) is an ongoing phase 2 randomized safety trial, in which ischemic stroke patients are randomly assigned to placebo or standard dose lovastatin versus short-term high-dose lovastatin.

### Propentofylline

Propentofylline acts by blocking the uptake of adenosine and inhibiting the PDE enzyme system (174, 175). Adenosine is released in response to cell damage after ischemic injury and acts on specific G-protein-coupled receptors on astrocytes, microglia, and infiltrating immune cells to regulate immune system function in the brain. Although the effects of adenosine protect neuronal integrity, adenosine might also aggravate neuronal injury by promoting inflammatory processes (176, 177). A detailed understanding of adenosine receptor function in the brain immune system should help researchers develop novel therapeutic strategies to treat HI-induced brain injury, which is associated with a dysfunctional immune response.

### Melatonin

In preclinical models of term brain HI, melatonin markedly decreased microglial activation in association with neuroprotection (178). A study of melatonin with therapeutic hypothermia in a piglet model of HI showed a substantial improvement compared with therapeutic hypothermia alone in preserving brain function measured by amplitude-integrated electroencephalogram, and reduced cell death in selectively vulnerable areas (179). There is an ongoing, prospective, double-blinded, randomized trial of premature newborns of less than 28 weeks’ gestational age for assessing neuroprotective role of melatonin in the United Kingdom (MINT trial, ISRCTN15119574).

### Epo

Epo and its receptor (EpoR) are expressed in the developing CNS and are required for normal brain development (180). Epo inhibits early mechanisms of brain injury by its anti-inflammatory, antiexcitotoxic, antioxidant, and antiapoptotic effects on neurons and oligodendrocytes (181–184). Currently, Epo is undergoing a trial of neuroprotection in preterm neonates (PENUT trial – preterm erythropoietin neuroprotection trial).

### Xenon

Xenon is an odorless, dense noble gas with anesthetic properties. Xenon’s neuroprotective properties have been demonstrated in cell culture (185), a rodent model of hypoxia-ischemia (186–190) and a neonatal pig model of global hypoxia-ischemia, wherein it can augment hypothermic neuropotection (191, 192). The precise mechanism of neuroprotection and any possible direct effects on inflammation remain to be explored.

### Delivery of anti-inflammatory and immunomodulator neuroprotective agents across BBB

Anti-inflammatory and immunomodulator agents like CBD are hydrophobic and, therefore, delivery across BBB becomes an important consideration for optimal neuroprotective effects within a safe dose-range. A novel approach is to use alternative delivery methods like nanoparticles, targeting the inflammatory system. Nanoparticles, such as polyamidoamine dendrimers have been shown to concentrate in activated microglia and astrocytes in the brains of newborn rabbits with cerebral palsy, but not healthy controls. This nanotechnology approach has shown excellent results to deliver dendrimer-bound *N*-acetyl-l-cysteine (NAC) to microglia to suppress neuroinflammation, using much lower concentrations than are needed with systemic dosing (193–195).

## Conclusion

Neuroinflammatory and neuroimmune dysregulation play a key role in secondary neuronal damage after global HI injury in the developing brain. Microglia activated by HI, initiate a cascade of inflammatory reactions that lead to neuronal damage. Indeed, a growing number of anti-inflammatory and immunomodulatory compounds have shown promising neuroprotective effects in preclinical settings. Some of these compounds have even entered clinical trials for adult victims of stroke. However, substantial work is needed to improve our understanding of neuroinflammation after global HI injury in developing brain. The specific issues yet to be determined in relation to inflammatory and immunologic mechanisms after global HI include the temporal, topographic, and gender pattern of neuroinflammation, neuronal–glial regulatory interactions, and their contribution to secondary neuronal injury, the role of the peripheral immune system in potentiating secondary brain injury after multi-organ ischemia, and the development of anti-inflammatory and immunomodulatory compounds as neuroprotective agents after HI injury of developing brain.

## Conflict of Interest Statement

The authors declare that the research was conducted in the absence of any commercial or financial relationships that could be construed as a potential conflict of interest.
